# Loss of PHD3 in myeloid cells dampens the inflammatory response and fibrosis after hind-limb ischemia

**DOI:** 10.1038/cddis.2017.375

**Published:** 2017-08-10

**Authors:** Angelika Beneke, Annemarie Guentsch, Annette Hillemann, Anke Zieseniss, Lija Swain, Dörthe M Katschinski

**Affiliations:** 1 Institute of Cardiovascular Physiology, University Medical Center, Göttingen, Germany

## Abstract

Macrophages are essential for the inflammatory response after an ischemic insult and thereby influence tissue recovery. For the oxygen sensing prolyl-4-hydroxylase domain enzyme (PHD) 2 a clear impact on the macrophage-mediated arteriogenic response after hind-limb ischemia has been demonstrated previously, which involves fine tuning a M2-like macrophage population. To analyze the role of PHD3 in macrophages, we performed hind-limb ischemia (ligation and excision of the femoral artery) in myeloid-specific PHD3 knockout mice (PHD3^−/−^) and analyzed the inflammatory cell invasion, reperfusion recovery and fibrosis in the ischemic muscle post-surgery. In contrast to PHD2, reperfusion recovery and angiogenesis was unaltered in PHD3^−/−^ compared to WT mice. Macrophages from PHD3^−/−^ mice showed, however, a dampened inflammatory reaction in the affected skeletal muscle tissues compared to WT controls. This was associated with a decrease in fibrosis and an anti-inflammatory phenotype of the PHD3^−/−^ macrophages, as well as decreased expression of Cyp2s1 and increased PGE2-secretion, which could be mimicked by PHD3^−/−^ bone marrow-derived macrophages in serum starvation.

Non-sterile as well as sterile inflictions of tissues can induce an inflammatory response.^1^ For both the innate immune system is the first line of defense, which is recruited to combat a potential threat. In the case of sterile inflammation the innate immune system supports tissue reconstitution. This has been implicated during the course of ischemic diseases such as myocardial infarction, stroke or peripheral arterial disease (PAD).^2, 3^ PAD is a devastating disorder with high rates of morbidity and mortality. Symptoms of PAD range from claudicatio intermittens to the loss of the inflicted extremity. Distal to the vessel occlusion an ischemia-induced inflammatory infiltrate develops in which neutrophils and macrophages are the key members.^4^ Hypoxia is a prominent feature of the inflammatory microenvironment due to increased oxygen demand and decreased supply. Hypoxia can actively affect inflammatory processes through the oxygen-sensitive regulation of the hypoxia-inducible factor (HIF) signaling pathway in multiple immune cell subtypes that are either resident within the inflamed tissue or have migrated from the oxygenated blood to the hypoxic inflammatory milieu.^5^ HIF comprises two subunits, that is, the oxygen dependently regulated HIF*α* subunit and the constitutively expressed HIF-1*β* subunit.^6^ At the molecular level, HIF*α* is regulated by three well described prolyl-4-hydroxylase domain enzymes (PHD) 1-3.^7^ Under consumption of molecular oxygen, HIFα is hydroxylated by the PHDs subsequently leading to pVHL-dependent ubiquitination and proteasomal degradation.^8^ In hypoxia, PHD-mediated hydroxylation is impaired by the lack of the co-substrate oxygen, which results in the stabilization of HIFα, heterodimerization with its *β*-subunit and activation of HIF target genes. Via those target genes, HIF induces metabolic adaptation, neovascularization but also shapes the ischemia-induced inflammation.^9^

There is clear evidence for differential roles of the PHD isoforms in immune cell subpopulations in both resting and activated states.^10^ Most of these analyses were performed in infectious disease models neglecting sterile inflammation. Further investigation is required to particularly understand the role of PHDs in sterile inflammation especially in light of the fact that hydroxylase inhibitors are tested for their tissue protective potential in case of ischemia-related diseases.^11^ Experimental hind-limb ischemia in mice, which is induced by femoral artery ligation, is a well-accepted disease model for studying the consequences of ischemia-induced inflammation, fibrosis, neovascularization and tissue reconstitution.^12^ Ubiquitious non-cell type-specific deletion of the PHDs in genetically modified mice or local depletion via injection of shRNAs in the ischemic muscles targeting the PHDs stabilize HIF-1*α*, promote neovascularization and improve perfusion in murine models of hind-limb ischemia.^13, 14^ Thus, inhibition of PHDs is a potentially effective option for advanced PAD. Most interestingly, Takeda *et al.*^15^ reported that deleting PHD2 cell type specifically in myeloid cells likewise increased neovascularization. The three PHD variants in mammalia differ regarding organ-specific expression, their inducibility in hypoxia and other non-HIF-related functions.^16^ Especially, PHD3 has been associated with cell survival decisions and altered macrophage function.^17^ A myeloid-specific knockout of PHD3 affects cell survival and apoptosis of neutrophils and macrophages.^18^ In the context of bacterial lung or intestinal infections, it has been demonstrated that a myeloid-specific PHD3 knockout results in hyperinflammation.^19, 20^ The consequences for a non-sterile ischemic inflammation, however, are unknown. Therefore, we analyzed the consequences of a hind-limb ischemia in myeloid-specific PHD3 knockout mice.

## Results

### Reperfusion and angiogenesis after hind-limb ischemia are unchanged in PHD3^−/−^ mice

**Figure 1:**
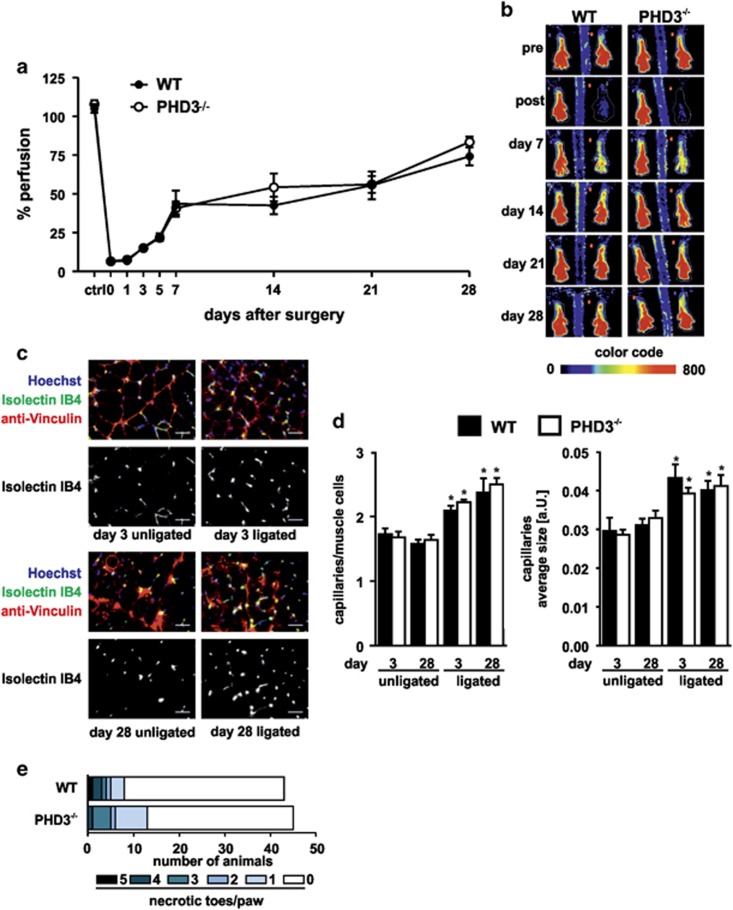
Perfusion recovery and angiogenesis after hind-limb ischemia are unchanged in PHD3^−/−^ mice. (**a**) Perfusion was measured from Laser Doppler images before, directly after and 1, 3, 5, 7, 21 and 28 days after hind-limb ischemia surgery. Groups included at least seven mice for each time point and each genotype. (**b**) Exemplary images of Laser Doppler measurements are shown; color code represents the intensity of perfusion (arbitrary intensity units). (**c**) Cryosections of the gastrocnemius muscle isolated from the ischemic (ligated) and non-ischemic control (unligated) leg were stained for isolectin IB4, vinculin and nuclei (Hoechst) to visualize capillaries and muscle cells. Scale bars represent 50 *μ*m. (**d**) The amount and average area of capillaries of sections of at least five mice per group were quantified using ImageJ. (**e**) The number of necrotic toes on the ligated legs was quantified after hind-limb ischemia surgery. Graphs show mean±S.E.M. with *P<0.05

Surgical ligation and excision of the femoral artery in mice induces severe ischemia distally to the occlusion in the GM of the calf including intense inflammatory processes. The inflammation is accompanied by ischemia-induced angiogenesis and muscle tissue remodeling. Arteriogenesis, which is induced by altered shear-stress is precluded by the hind-limb ischemia model applied in our study as the pre-existing caudal femoral artery (A. profunda femoris) was excised. Activated macrophages contribute angiogenic and angiostatic factors in the process of ischemia-induced angiogenesis and thus perfusion recovery. The overall impact of angiogenesis and thus hind-limb perfusion can be assessed by LDPI of the feet, which also allows sequential analysis of the reperfusion capacity in one mouse. Accordingly, we performed LDPI with WT and PHD3^−/−^ mice up to 28 days after hind-limb ischemia surgery (Figures 1a and b). In both genotypes, perfusion was significantly impaired below 10% compared to the non-ligated leg in response to femoral artery ligation and recovered to around 70% on day 28. Neither the time course nor the extent of reperfusion recovery was significantly different between WT and PHD3^−/−^ mice. The unaltered angiogenic response in the PHD3^−/−^ mice was additionally verified by immunohistochemistry analysis of capillaries by Isolectin IB4 tracing of endothelial cells and co-stainings with Hoechst (nuclei stain) and anti-vinculin antibodies in the ischemic GM muscles on day 3 and 28 after hind-limb ischemia (Figures 1c and d). The number and the size of the capillaries were significantly increased on both days in the ligated compared to the unligated leg demonstrating that the induced ischemia indeed triggered an angiogenesis response, which however was unaltered comparing WT and PHD3^−/−^ mice. This was likewise reflected in the analysis of necrotic toe development in consequence of the ischemic tissue destruction. Mice from both genotypes developed necrotic toes with no difference in the extent or number of necrotic toes per paw (Figure 1e).

### Macrophage infiltration in the ischemic muscle is altered in PHD3^−/−^ mice

**Figure 2:**
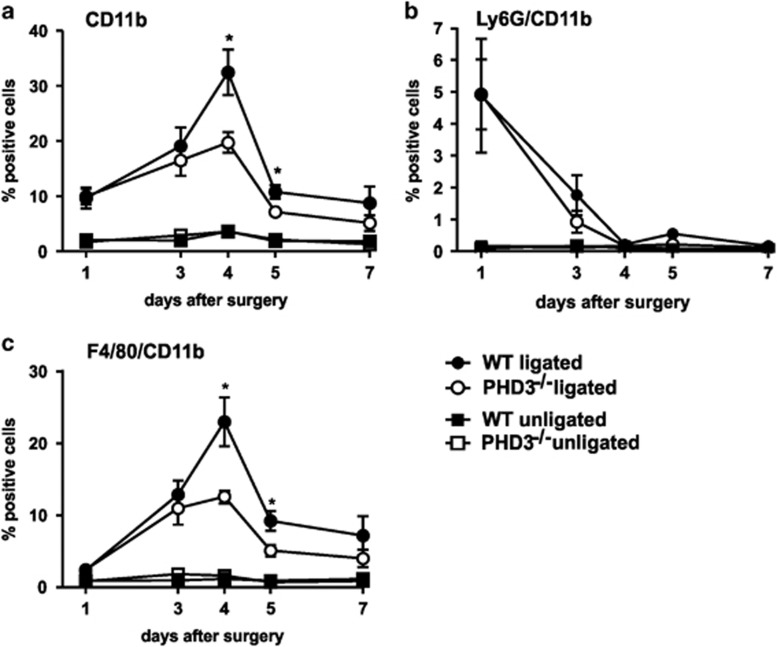
PHD3^−/−^ mice show a dampened leukocyte infiltration into the ischemic muscle after hind-limb ischemia. Leukocyte infiltration was analyzed via flow cytometry over the course of 7 days after surgery within the gastrocnemius muscle of the ischemic (ligated) and non-ischemic control (unligated) legs of wild type (WT) and PHD3^−/−^ mice. Leukocytes were identified as CD11b-positive cells (**a**), whereas neutrophils were identified as Ly-6G CD11b-positive cells (**b**) and macrophages as F4/80 CD11b-positive cells (**c**). At least five mice were analyzed per group. Graphs represent mean±S.E.M. with *P<0.05

Immediately after an ischemic injury, the innate immune system is activated and is responsible for recruitment of inflammatory cells from the circulation. We quantified the inflammatory infiltrate in the GM in the ischemic *versus* non-ischemic legs of WT and PHD3^−/−^ mice 1–7 days after hind-limb ischemia by CD11b staining and FACS analysis (Figure 2a). The leukocyte infiltrate was significantly increased 1 day after hind-limb ischemia, peaked at day 4 and was almost completely resolved on day 7 with no kinetic difference in WT and PHD3^−/−^ mice. The number of CD11b-positive cells, however, was significantly lower in the PHD3^−/−^ mice. CD11b is an integrin family member and is expressed on the surface of many leukocytes including monocytes, neutrophils, natural killer cells, granulocytes, and macrophages. A sequential influx of neutrophils and macrophages upon injury is a hallmark of acute tissue damage. Neutrophils are the first line of defense; macrophages are subsequently needed to remove the invading neutrophils that undergo NETosis or apoptosis. Characterization of the infiltrating leukocytes by Ly-6G and F4/80 – CD11b co-staining confirmed the sequential activation of neutrophils and macrophages peaking at day 1 and day 4, respectively (Figures 2b and c). Although the infiltration with neutrophils was unaltered in WT compared to PHD3^−/−^ mice, the number of macrophages present in the ischemic muscle was significantly lower in the PHD3^−/−^ mice, demonstrating that mainly macrophages are responsible for the difference in the CD11b-positive inflammatory infiltrate.

**Figure 3:**
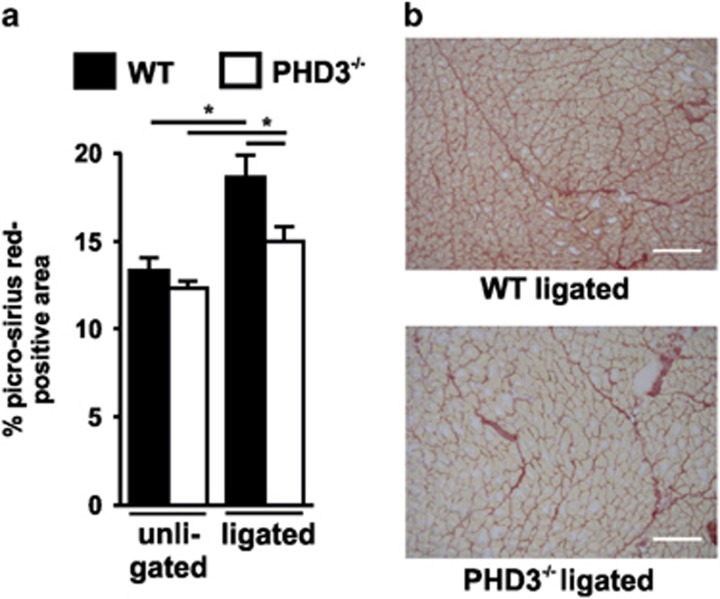
PHD3^−/−^ mice show less fibrosis after hind-limb ischemia. Fibrosis was visualized using picro-sirius red staining on cryosections of gastrocnemius muscles 28 days after surgery (**a**). The picro-sirius red positive area was quantified via ImageJ (**b**). seven mice per group were analyzed. Scale bars represent 200 *μ*m. Graphs show mean±S.E.M. with * *P*<0.05

Infiltrating macrophages have a major role in cellular and molecular events of fibrosis. Scarring is necessary to address the loss of tissues that cannot rapidly or only incompletely regenerate. A persistent accumulation of macrophages can prolong tissue inflammation and aggravate fibrosis, which is associated with a possible long-term loss of tissue function. The extent of fibrosis in the ischemic muscle after hind-limb ischemia was therefore analyzed (Figures 3a and b). In line with the lower numbers of infiltrating macrophages, we observed significantly less fibrosis in the ischemic GM of the PHD3^−/−^ compared to WT mice.

### PHD3^−/−^ macrophages in the ischemic muscle have an anti-inflammatory phenotype

**Figure 4:**
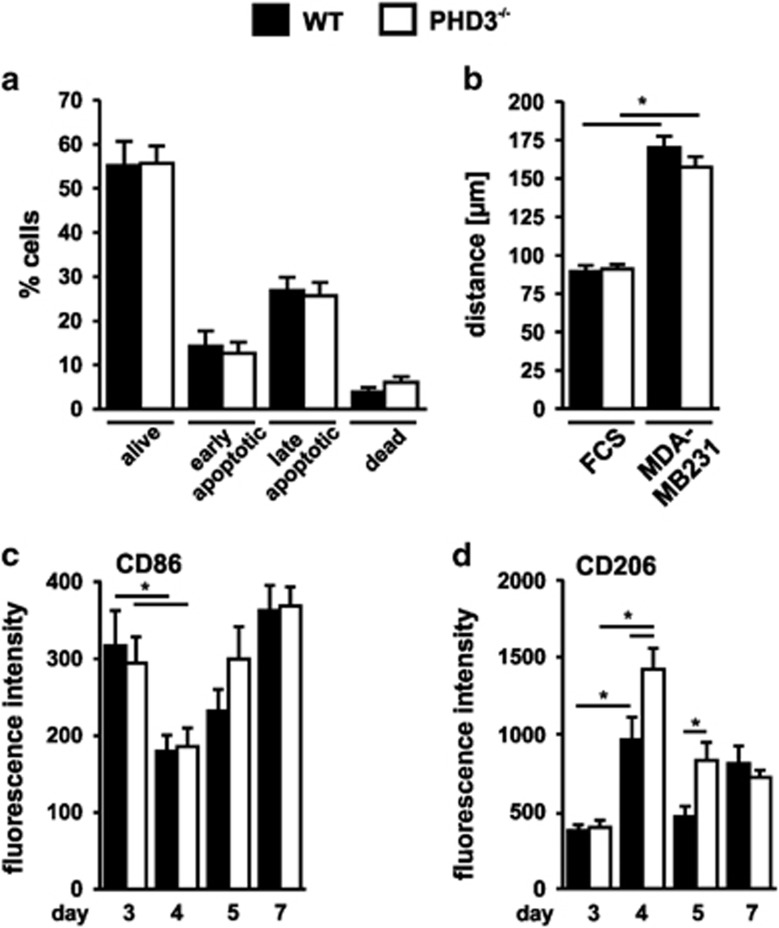
PHD3^−/−^ macrophages are more M2-polarized after hind-limb ischemia surgery compared to wild type (WT) and show unaltered apoptosis and migratory behavior. (**a**) Apoptosis was analyzed in F4/80 CD11b-positive macrophages isolated from the gastrocnemius muscles (GM) of WT and PHD3^−/−^ mice 4 days after hind-limb ischemia via stainings with Zombie green and Annexin V. Dead cells were identified as Annexin V− Zombie green high, late apoptotic cells as Annexin V+ Zombie green high, early apoptotic cells as Annexin V+ Zombie green low and living cells as Annexin V− Zombie green low cells. At least six animals were included per genotype. (**b**) The migratory capacity of BMDM was analyzed in a single-cell migration assay via the accumulated distance cells traveled within 6 h. The graph is representative for at least three independent experiments. F4/80 CD11b-positive macrophages isolated from the GM after surgery were analyzed for their geometric mean fluorescence intensity of CD86 (**c**, M1-marker) and CD206 (**d**, M2-marker). At least five animals were included per genotype. Graphs show mean±S.E.M. with *P<0.05

Changing tissue environments, which occur after the onset of tissue injury, shape the phenotype of macrophages to provide them with additional functional properties like altered life span or pro- and anti-inflammatory characteristics. This helps to meet the tissues need in addressing the damage. As a defining hallmark of macrophages, this plasticity allows them to adjust their effector functions. As PHD3 has been described to alter viability of myeloid cells, we analyzed markers for apoptosis and necrosis in the macrophages isolated from the inflamed muscles after hind-limb ischemia. We found a significant proportion of macrophages with markers characteristic for late apoptosis, however, without any difference when comparing WT and PHD3^−/−^ macrophages (Figure 4a). In a previous report, we have demonstrated that a knockout of PHD2 in macrophages results in a HIF-mediated metabolic phenotype associated with impaired migration and phagocytosis, which might result in a delayed inflammatory infiltrate.^21^ A similar migratory impairment of the PHD3^−/−^ macrophages was excluded in single-cell migration experiments (Figure 4b).

The existence of a plethora of macrophage polarization states is critical for the adequate onset, regulation, and resolution of inflammatory responses.^22^ M1- and M2-polarized macrophages are the extremes of a continuum of intermediate cells, which can be seen after stimulation of macrophages with certain cytokines *in vitro*. *In vivo* macrophages are more or less skewed into the direction of one of these extremes. We analyzed the presence of CD68 and CD206 by FACS analysis as characteristic markers for M1- and M2-like polarized cells, respectively, on CD11b/F4/80-positive cells in the ischemic GM (Figures 4c and d). Similar to the kinetic of the macrophage infiltrate, CD206 expression peaked on day 4 with an inverse pattern in expression of CD68. In line with the dampened inflammatory infiltrate in the PHD3^−/−^ mice after hind-limb ischemia, PHD3^−/−^ macrophages displayed significantly higher expression of CD206 on day 4 and 5 compared to WT macrophages supporting an anti-inflammatory phenotype.

### Differential expression of Cyp2s1 in PHD3^−/−^ macrophages

**Figure 5:**
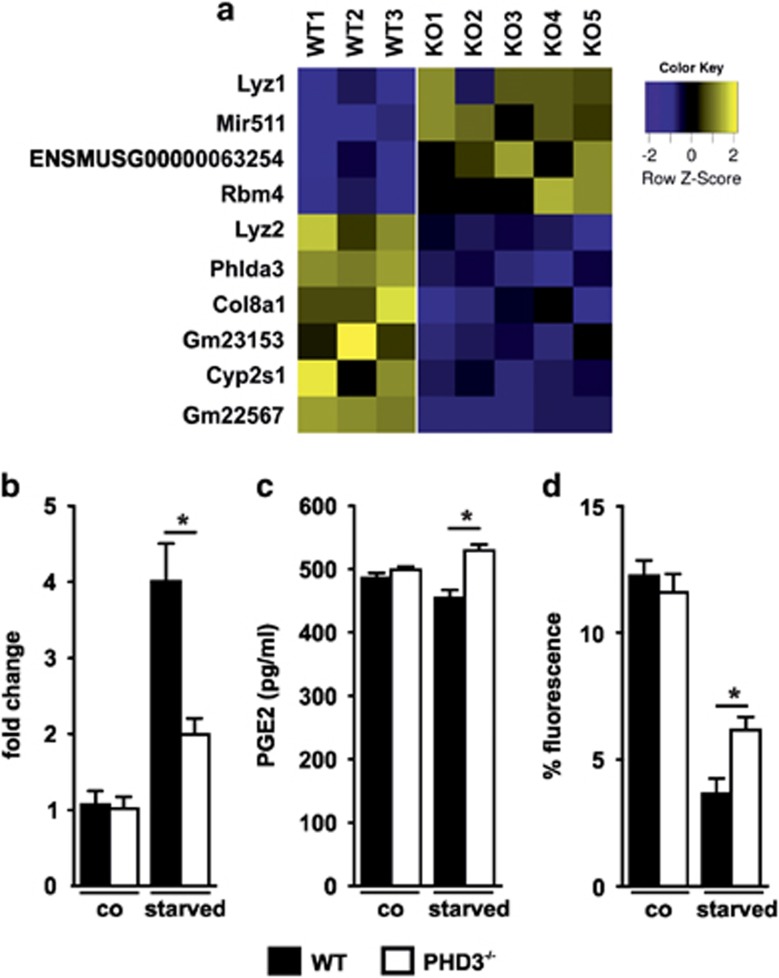
PHD3^−/−^ macrophages show a decreased expression of the cytochrome P450 enzyme Cyp2s1, which coincides with a higher PGE2-secretion and an increased phagocytotic capacity in PHD3^−/−^ BMDM upon starvation. (**a**) F4/80 CD11b-positive macrophages were sorted from gastrocnemius muscles of three wild type (WT) and five PHD3^−/−^ (KO) mice 5 days after hind-limb ischemia surgery and analyzed via RNA-sequencing. In total, four genes were significantly upregulated and six significantly downregulated in PHD3^−/−^ macrophages (log-fold change of at least±1). (**b**) RNA-expression of Cyp2s1 was analyzed in WT and in PHD3^−/−^ bone marrow-derived macrophages (BMDM) upon serum starvation (24 h). (**c**) Prostaglandin E2 was determined by ELISA in the supernatants of WT and PHD3^−/−^ BMDM (**d**) Phagocytosis of WT and PHD3^−/−^ BMDM in control conditions or after 24 h serum starvation. Graphs are representative for at least three independent experiments. Bars show mean±S.E.M. with *P<0.05

Understanding of the macrophage population switch is an attractive target to manipulate inflammation and to reduce fibrosis and scarring, which would improve healing and long-term tissue function. To gain insight into the differential inflammatory response of WT and PHD3^−/−^ macrophages, we FACS-sorted CD11b-F4/80-positive cells from the ischemic GM of 3 WT and 5 PHD3^−/−^ mice on day 5 after hind-limb ischemia, isolated RNA and performed RNA-seq analysis. The bioinformatic analysis revealed in total 10 differentially expressed RNAs (Figure 5a). The RNA-seq protocol included an mRNA fragmentation approach prior to sequencing to gain sequence coverage of the whole transcript. The total number of reads for a given transcript is thus proportional to the expression level of the transcript multiplied by the length of the transcript, which is reflected in the reads per kilobase transcript per million (RPKM). Of note, the cut-off chosen (base mean >50) for analysis did not allow PHD3 to appear in the list of differentially expressed candidates based on its low overall expression level reflected by a base mean of 18 and RPKM value below 1. Among the identified candidates miRNA511 and Cyp2s1 were previously described to be associated with inflammatory functions. Although the differential expression of miRNA511 could not be verified in *in vitro* differentiated BMDM cultured in normoxia and in simulated ischemic conditions, that is, serum starvation (data not shown), the differential expression of Cyp2s1 was also seen in the cultured macrophages (Figure 5b). Interestingly, the difference was only detectable in serum starvation demonstrating that the PHD3-mediated differential expression is only applicable to those conditions. Cyp2s1 is a member of the cytochrome P (Cyp)450 superfamily of enzymes. Cyp2s1 is highly expressed in epithelial tissues of the respiratory, gastrointestinal, urinary tracts, and skin as well as in leukocytes of the monocyte/macrophage and lymphocyte series.^23^ Extra hepatic CYP epoxygenases can utilize endogenous substrates such as arachidonic acid, linoleic acid, eicosapentaenoic acid and docosahexenoic acid to generate bioactive lipid mediators.^24^ In this context, Cyp2s1 is an important enzyme in the metabolism of COX-derived prostaglandins including PGE2 at nanomolar concentrations, and thus has an important role in modulating inflammatory processes including macrophage phagocytosis. We verified that PGE2 expression and phagocytosis were indeed affected in the PHD3^−/−^ macrophages (Figures 5c and d). We found elevated Cyp2s1 expression in serum starvation. This response was significantly blunted in the PHD3^−/−^ BMDM. In line with the increased expression of Cyp2s1 in serum-starved WT BMDM, PGE2 levels were decreased in this condition. In contrast, the PGE2 levels in the PHD3^−/−^ BMDM were significantly higher in line with the Cyp2s1 response in these cells. In the early phase of inflammation, PGE2 can attract neutrophils and then serves as pro-inflammatory molecule.^25^ Most importantly, however, PGE2 produced by macrophages affects their function towards an anti-inflammatory tissue reconstituting phenotype.^26^ In line, phagocytosis of fluorescently labeled beads was increased after serum starvation.

## Discussion

Therapeutic angiogenesis is considered as a potential strategy for treating hind-limb ischemia.^27^ However, an efficient induction of mature and functional blood vessels remains still challenging. In previous reports, it was demonstrated that PHD2 expressed in macrophages is able to influence therapeutic angiogenesis after hind-limb ischemia.^15^ In sharp contrast, no similar findings were obtained in our study regarding PHD3 expressed in myeloid cells. Neither perfusion nor capillary number or average capillary size was altered in the PHD3^−/−^ compared to the WT mice after hind-limb ischemia. This is in line with the previous notion that the three PHD variants in mammalia differ regarding cell type-specific expression but especially regarding function. A compensatory mechanism by PHD3, which limits the HIF response as a consequence of a PHD2 knockout has been described in several cell and animal models.^21, 28, 29, 30^ This might explain why some phenotypes induced by a PHD2 knockout are more severe in combination with knocking out PHD3. A singular knockout of PHD3 alone, however, seems to reveal the PHD3 isoform-specific functions and might explain, why the phenotype observed in the PHD3^−/−^ compared to the WT mice are seen just in the ischemic legs *in situ* or under simulated ischemic conditions *in vitro*.

PHD3 has been associated with cell survival decisions and the course of inflammation.^31^ Moreover, PHD3 has been shown to cause apoptosis in neuronal cells and in cancer cells when expressed at high levels.^32, 33, 34, 35^ Compared to PHD2, PHD3 has been suggested to have non-HIF targets and downstream effectors.^36^ We found in the PHD3^−/−^ mice a dampened inflammatory infiltrate in the ischemic muscle, which contained a lower number of macrophages whereas the number of neutrophils was unaffected. The lower number of macrophages was not due to increased cell death as the number of apoptotic and dead macrophages were not significantly altered in the PHD3^−/−^ ischemic legs. However, the macrophage population switched to a more anti-inflammatory type. This was reflected by the increased expression of the M2-marker CD206 as well as the decreased expression of Cyp2s1. Bui *et al.* identified that human Cyp2s1 is an important enzyme in the metabolism of COX-derived prostaglandins at nanomolar concentrations, and the authors suggested that Cyp2s1 may have an important role in modulating the inflammatory process.^37, 38^ In macrophages Cyp2s1 is an epoxygenase, which has been described to metabolize PGG2 and PGH2 to 12-HHT and thereby influences PGE2 production.^39^ PGH2 is synthesized from arachidonic acids by the action of cyclooxygenase-1 and 2 via the intermediate product PGG2. PGH2 also serves as a substrate for PGE2 synthesis via the PGE2 synthases.^40^ PGE2 is a potent pro- and anti-inflammatory mediator, which in the early inflammatory phase mediates attraction and activation of neutrophils, but has also been described to enhance M2-polarization of macrophages and to interfere with inflammasome activation in sterile inflammation.^26, 41^ Cyp2s1 and PGE2 have both been described to affect inflammatory macrophage function like phagocytosis. Downregulation of Cyp2s1 by siRNA led to increased phagocytosis in monocytes.^39^ This is in line with the increased phagocytosis in PHD3^−/−^ BMDM found in our study. Most interestingly the effects of altered Cyp2s1 expression, PGE2 production and altered phagocytosis were seen in the BMDM under starved conditions only. In contrast to PHD1 and PHD2, PHD3 is highly inducible in hypoxia. Aside from this, PHD3 is also upregulated in other (patho)physiological conditions like vascular tissue injury,^42^ ageing ^43^ and especially nerve growth factor removal.^32, 33, 34, 35, 36, 37, 38, 39, 40, 41, 42, 43, 44^ In neural cells, PHD3 is required and is even sufficient to induce apoptosis after growth factor removal. Serum starvation as employed in our study might mimic at least in part the *in vivo* situation of the macrophages in the tissue after hind-limb ischemia. Although we can formally not delineate, whether the role of PHD3 for Cyp2s1 expression *in vitro* in consequence of serum starvation is mainly due to the fact, that these conditions foster PHD3 expression or whether additional factors are induced, these experiments demonstrate that studies, which rely on BMDM under standard cell culture conditions only, might not be able to reveal PHD3-related functions fully. Some features might just uncover in the *in vivo* situation mediated by the interplay with other immune cells or special tissue conditions like nutrient depletion. This applies to the identified anti-inflammatory Cyp2s1 expression profile and phagocytosis observed in the PHD3^−/−^ macrophages. Overall, the anti-inflammatory phenotype in the ischemic leg in the PHD3^−/−^ mice was associated with a decrease in fibrosis.

Small molecule inhibitors of the PHD enzymes are explored regarding their use in the context of ischemic diseases.^16^ The inhibitors applied so far, are not selective regarding PHD isoforms. However, it is worthwhile to know, which isoform is mediating tissue protective function and which cell types are involved. According to our data, inhibition of PHD3 activity in macrophages might not affect therapeutic angiogenesis it might however indeed affect the non-sterile inflammation.

## Materials and methods

### Mice

Generation of LysM Cre+/− x PHD3fl/fl (PHD3^−/−^) mice and LysM Cre^−/−^ × PHD3^fl/fl^ (WT) littermate control mice were described previously.^18^ Animals were maintained on a C57BL/6 background.

### Hind-limb ischemia

All animal experiments were performed according to the German animal protection law and approved by the Niedersächsische Landesamt für Verbraucherschutz und Lebensmittelsicherheit in Oldenburg (animal experimentation numbers 33.942502-04-16/2139 and 33.9-42502-04-11/0413). Unilateral hind-limb ischemia was performed as described by Limbourg *et al.*^12^ In brief, male mice at the age of 8–12 weeks were anaesthetized by intraperitoneal injection of 15 mg/kg body weight xylazin, 75 mg/kg body weight ketamine in 0.9% sodium chloride and placed on a 37 °C heating pad. Through two longitudinal incisions above the knee and at the groin the A. femoralis was exposed and occluded proximal and distal to the A. profunda femoris with silk sutures using triple surgical knots. The part of the artery between the knots was removed without damaging the femoral vein and nerve and the skin was sutured. This surgery treatment enables the induction of severe ischemia in the M. gastrocnemius (GM) and analysis of the resulting angiogenesis, but precludes arteriogenesis development by pre-existing collateral arterioles in the skeletal muscles of the thigh.

### Laser doppler perfusion imaging

Blood perfusion was measured using PeriScan PIM3 laser Doppler (Perimed Instruments, Cologne, Germany). For the scanning procedure mice were anaesthetized by intraperitoneal injection of 15 mg/kg body weight xylazin, 75 mg/kg body weight ketamine in 0.9% sodium chloride and placed on a 37 °C heating pad. Perfusion rate was analyzed using the PIMsoft 1.3 software (Perimed Instruments, Cologne, Germany) by comparing the perfusion units, calculated from the heat map of defined regions of interest of the ligated *versus* non-ligated leg.

### Analysis of capillary density and size

Cryosections were fixed for 20 min in a 1:1 acetone/methanol solution at −20 °C. Incubation in blocking solution (10% normal goat serum, 0.1% Triton X in PBS) was followed by staining of the sections with 1:200 Alexa488-coupled isolectin GS-IB4 (I21411, Invitrogen, Darmstadt, Germany) and 1:100 anti-vinculin antibodies (V9264, Sigma, St. Louis, MO, USA). After extensive washing with PBS, slides were incubated with 1:200 TR-coupled anti-mouse antibodies (Sc-2781, Santa Cruz Biotechnology, Santa Cruz, CA, USA). Sections were washed in PBS and stained using 1:500 Hoechst (Thermo Scientific, Waltham, MA, USA) for 10 min. Subsequently, samples were embedded in Mowiol (3% glycerin, 1.33% Mowiol, 0.133 Tris pH 8.5, 0.1% DABCO). Stained sections were imaged by fluorescence microscopy (Zeiss Observer D1 microscope, Carl Zeiss, Göttingen, Germany). 15–20 images were taken of different areas of the muscle. The amount and average size of capillaries were then analyzed using ImageJ (U.S. National Institutes of Health, Bethesda, MD, USA).

### Picro-sirius red staining

Cryosections of GM were placed in xylol and subsequently in 99-60% ethanol. Slides were incubated in 0.1% Picro-sirius red staining solution (Sigma) for 60 min. After washing with 1% acetic acid, sections were incubated in ethanol and xylol and embedded in Roti-Histo-kitt (Roth). The whole section was imaged (Zeiss Observer D1 microscopy) and analyzed for Picro-sirius red positive areas using ImageJ.

### Isolation and differentiation of bone marrow-derived macrophages

Bone marrow-derived macrophages were isolated and differentiated as described previously.^18^ Resulting cell suspension was cultivated for 48 h. Purity of the adherent macrophages were analyzed by F4/80 and CD11b staining and FACS analysis.

### Hypoxic incubation

For culturing cells in defined hypoxic conditions (1% O_2_) an *in vivo* Hypoxia workstation (Ruskinn Technologies, Bridgend, South Wales, UK) was used.

### Single cell migration

Migration capacity was determined using a single-cell migration approach, which studies random migration as described before.^21^

### Macrophage viability

To quantify viability of the FACS analyzed macrophages in the digested GM, F4/80 CD11b double-positive cells were stained with Pacific blue coupled Annexin V (1:50, Biolegend) and FITC coupled Zombie Green (1:1000, Biolegend, San Diego, CA, USA). Annexin V-/Zombie Green low, Annexin V+/Zombie Green low, Annexin V+/Zombie green high and Annexin V-/Zombie Green high F4/80+CD11b+ cells were regarded to be viable, early apoptotic, late apoptotic and dead, respectively.

### FACS analysis

GM were removed from the legs of mice, cut into pieces, which were suspended in digestion buffer (450 U/ml collagenase IV, 1.2 U/ml dispase II in PBS) and incubated for 45 min at 37 °C with vigorous pipetting every 15 min. After stopping the digestion with DMEM containing 15% FCS, 1% p/s samples were filtered using a 40 *μ*m mesh, centrifuged, washed in PBS and counted. Overall, 300 000-500 000 cells per sample were used for further analysis. Non-specific Fc binding sites were blocked with Mouse BD Fc Block (Biolegend). For analysis of cell subpopulations fluorescently coupled antibodies targeting the following surface markers were added, neutrophils Ly-6G-FITC, CD11b-PE/Cy7; macrophages F4/80-FITC, CD11b-PE/Cy7; M1 polarization F4/80-FITC, CD11b-PE/Cy7, CD86-PE; M2-polarization F4/80-FITC, CD11b-PE/Cy7, CD206-APC. All antibodies and their respective isotype controls were purchased from Biolegend. Stained cells were analyzed using the FACS Canto II flow cytometer (BD Biosciences, Heidelberg, Germany). A compensation was calculated for all staining protocols.

### RNA isolation and qRT-PCR

RNA was isolated using TRIzol (Thermo Fisher Scientific, Waltham, MA, USA). RNA was transcribed into cDNA using the First strand cDNA synthesis kit (Thermo Fisher Scientific). qRT-PCR was performed using the SensiMix SYBR Low-ROX kit according to the manufacturer’s instructions. The following primers were applied: Cyp2s1: 5′-TGCTCCTGCTGAGATACCCT-3′ and 5′-GAAGCAAGTGGTCCTCGTGA-3′, S12: 5′-GAAGCTGCCAAGGCCTTAGA-3′ and 5′-AACTGCAACCAACCACCTTC-3′, PHD3: 5′-GGCCGCTGTATCACCTGTAT-3′ and 5′-TTCTGCCCTTTCTTCAGCAT-3′

### Transcriptome analysis

GM lysates were washed and then stained with the fluorescently labeled F4/80-PE and CD11b APC antibodies. Doublets were discriminated using SSC-A *versus* SSC-W gating. The cell population positive for both markers were selected and sorted via a FACS Aria III flow cytometer (BD Biosciences, Heidelberg, Germany) into 1 ml TRIzol. RNA was isolated using chloroform/isopropanol. RNA was prepared for sequencing using the TruSeq total RNA sample preparation kit (Illumina, San Diego, CA, USA). This includes rRNA depletion, fragmentation of RNA and synthesis of cDNA. Modified cDNA was amplified in a PCR reaction and sequenced using the Illumina technique. The results of the total RNA-sequencing were analyzed using DeSeq2 software (Illumina, San Diego, CA, USA). Only genes that showed a significant log2 fold change of more than 1 or less than −1 were considered as candidate RNAs. Statistical significance was determined using the Benjamin–Hochberg correction. Adjusted *P*-values <0.05 were considered statistically significant.

### Prostaglandin E2 ELISA

Prostaglandin E2 (PGE2) levels in cell culture supernatants were measured using a chemiluminescent ELISA kit according to the manufacturer’s recommendation (Abcam, Cambridge, UK).

### Phagocytosis

Phagocytotic capacity was analyzed using fluorescently labeled beads as described previously.^21^

### Statistics

Statistical significance between the two different genotypes was calculated by using an unpaired two-tailed student’s *t*-test. A significant difference between two means was defined by a *P*-value <0.05.
